# Epidemiology of Resistance Determinants Identified in Meropenem-Nonsusceptible *Enterobacterales* Collected as Part of a Global Surveillance Study, 2018 to 2019

**DOI:** 10.1128/aac.01406-22

**Published:** 2023-04-19

**Authors:** Mark Estabrook, Astrid Muyldermans, Daniel Sahm, Denis Pierard, Gregory Stone, Eric Utt

**Affiliations:** a IHMA, Schaumburg, Illinois, USA; b Department of Microbiology and Infection Control, Vrije Universiteit Brussel, Universitair Ziekenhuis Brussel, Brussels, Belgium; c Pfizer Inc., Gorton, Connecticut, USA

**Keywords:** carbapenemase, *Enterobacterales*, carbapenem-nonsusceptible, Enterobacterales

## Abstract

The objective of this study was to describe the frequency of resistance determinants in meropenem-nonsusceptible (MEM-NS) *Enterobacterales* isolates collected in 2018 and 2019 as a part of the ATLAS global surveillance program. Among a total of 39,368 *Enterobacterales* isolates collected in 2018 and 2019, 5.7% were MEM-NS (MIC ≥2 μg/mL). Among the different regions, the proportion of MEM-NS isolates ranged from 1.9% (North America) to 8.4% (Asia/Pacific). The majority of MEM-NS isolates collected were of the species Klebsiella pneumoniae (71.5%). Among the MEM-NS *Enterobacterales* isolates collected, metallo-β-lactamases (MBL) were identified in 36.7%, KPC in 25.5%, and OXA-48-like in 24.1%. The predominance of resistance mechanisms among MEM-NS isolates varied by region: MBLs were dominant in isolates collected in Africa and Middle East (AfME, 49%) and Asia/Pacific (59.4%), OXA-48-like carbapenemases were predominant in Europe (30%), and KPC in Latin America (51.9%) and North America (53.6%). NDM β-lactamases accounted for the majority of MBLs identified (88.4%). Of the 38 carbapenemase variants identified, NDM-1 (68.7%), KPC-2 (54.6%), OXA-48 (54.3%), and VIM-1 (76.1%) were the common variants within their respective families. Among the MEM-NS isolates, 7.9% co-carried two carbapenemases. Notably, the proportion of MEM-NS *Enterobacterales* increased from 4.9% in 2018 to 6.4% in 2019. The results of this study show a continuation of the trend of increasing carbapenem-resistance within clinical *Enterobacterales* with mechanisms of resistance varying across different regions. The existential threat to public health posed by the continued spread of nearly untreatable pathogens requires a multifaceted approach to prevent the collapse of modern medicine.

## INTRODUCTION

*Enterobacterales* are a family of Gram-negative bacteria, including a wide array of clinically relevant species such as Escherichia coli, Klebsiella spp., and Enterobacter spp. that are also the most frequent species encountered in hospital intensive care units and are responsible for community- and hospital-acquired infections (1, 2). Carbapenems are broad-spectrum antibiotics that have been widely used to treat infections caused by multidrug-resistant Gram-negative bacteria, including *Enterobacterales* (3). With the increased use of carbapenems, worldwide emergence of carbapenem-resistant *Enterobacterales* (CRE) has been reported, which in clinical settings have limited treatment options and are associated with increased morbidity and mortality (1, 3–6). The continued surveillance of antimicrobial resistance determinants is one critical arm of the multifaceted approach needed to effectively combat ever-more resistant infectious organisms.

Resistance to carbapenems among CREs is primarily achieved by production of carbapenemases capable of hydrolyzing carbapenems and most other β-lactams, although hyperproduction AmpC combined with mutations of porins can be involved as well (6–8). Carbapenemases produced by *Enterobacterales* have been identified among three out of four Ambler classes of β-lactamases—class A, class B, and class D. The most notable among class A enzymes include KPC and some variants of GES, while class D carbapenemases are most often those closely related to OXA-48. Both class A and D enzymes have serine residues at their active sites. Ambler class B enzymes are metallo-β-lactamases (MBLs) and have metal ion cofactors in their active sites (9). The most widespread MBLs among clinical *Enterobacterales* isolates include NDM, IMP, and VIM (1, 3, 6). The spectrum of activity differs across enzymes of the different classes; KPCs hydrolyze all clinically available β-lactam agents but, can be inhibited by diazabicyclooctane β-lactamase inhibitors, MBLs hydrolyze all clinically available β-lactam agents except aztreonam, and are insensitive to clinically available β-lactamase inhibitors (BLIs), and OXA-48-like enzymes which generally hydrolyze penicillins and carbapenems, and are poorly inhibited by available BLIs (6). The OXA-48-type β-lactamases are now routinely encountered in infections caused by carbapenem-resistant *Enterobacterales*. These enzymes are of high and growing clinical significance due to the importance of carbapenems in treatment of health care-associated infections by Gram-negative bacteria, the wide and increasing dissemination of OXA-48 enzymes on plasmids, and the challenges posed by their detection (10).

A previous study assessed the distribution of β-lactamase resistance determinants in meropenem-nonsusceptible *Enterobacterales* among isolates collected between 2012 and 2017 (7). The current study is a continuation of the previous study with the objective of describing the epidemiology of β-lactamase resistance determinants in meropenem-nonsusceptible (MEM-NS) *Enterobacterales* among isolates collected in 2018 and 2019.

## RESULTS

**FIG 1:**
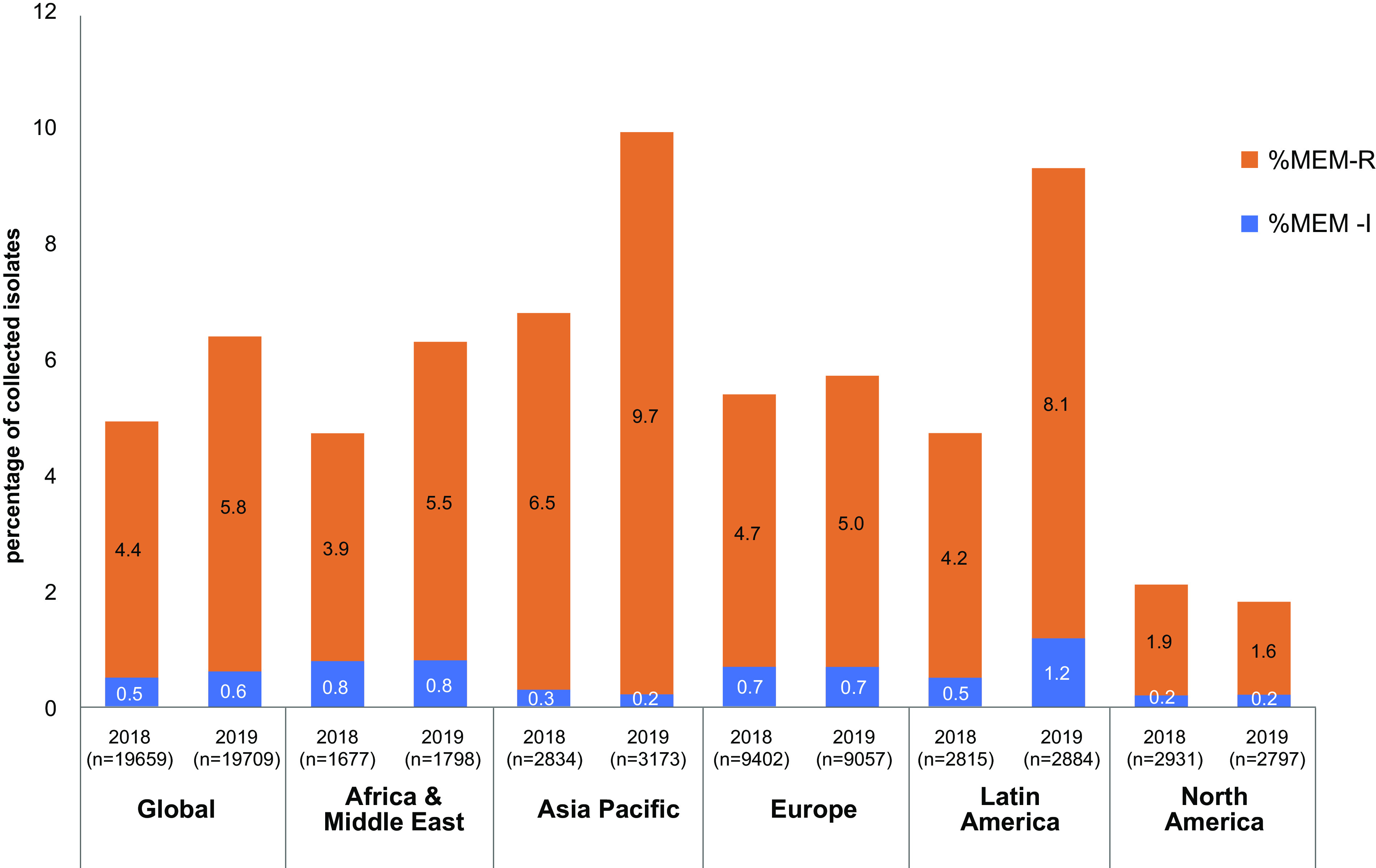
Distribution of meropenem-nonsusceptible *Enterobacterales* collected globally and across different regions in 2018 and 2019. MEM-R, meropenem-resistant; MEM-I, meropenem-intermediate; n, total number of isolates collected. Countries contributing isolates from different regions included Israel, Jordan, Kuwait, Morocco, Nigeria, Qatar, Saudi Arabia, South Africa (Africa and Middle East); Australia, Hong Kong, India, Japan, South Korea, Malaysia, Philippines, Singapore, Taiwan, Thailand (Asia Pacific); Belgium, Croatia, Czech Republic, France, germane, Greece, Hungary, Ireland, Italy, Latvia, Lithuania, Netherlands, Poland, Portugal, Romania, Russia, Spain, Switzerland, Turkey, Ukraine, United Kingdom (Europe); Argentina, Brazil, Chile, Columbia, Costa Rica, Guatemala, Mexico, Panama, Venezuela (Latin America); Canada and United States (North America).

**FIG 2:**
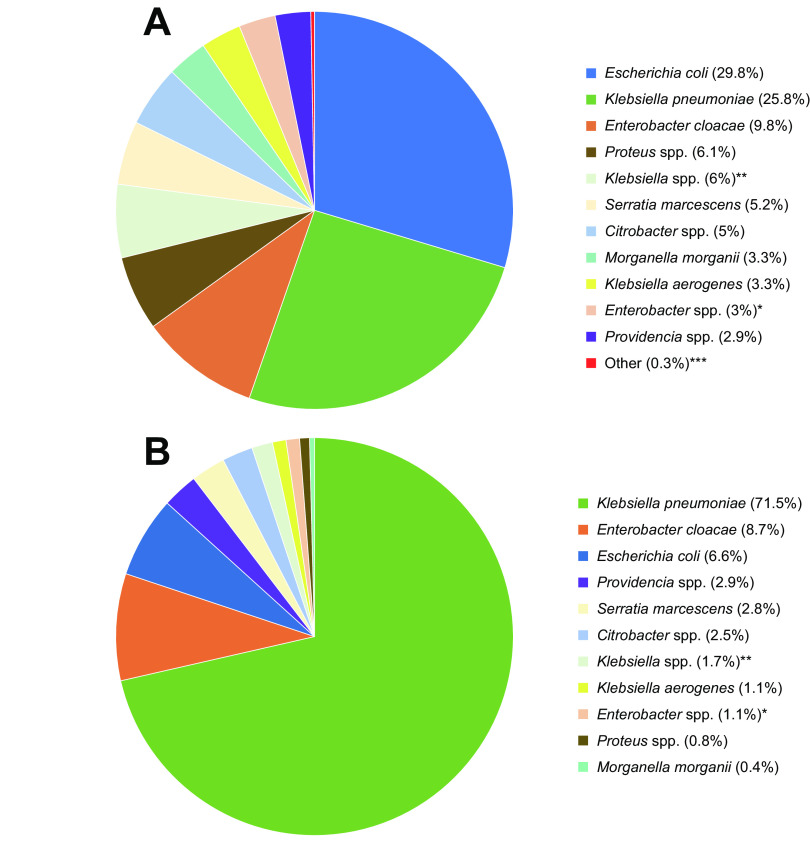
Distribution of all organisms and meropenem-nonsusceptible organisms among *Enterobacterales* isolates collected globally in 2018 and 2019. (A) Distribution of isolates of all organisms among *Enterobacterales* (N = 39,368). *Does not include isolates of E. cloacae; **Does not include isolates of K. aerogenes and K. pneumoniae; ***Includes *Cronobacter* spp. Escherichia vulneris, Hafnia alvei, Kosakonia cowanii, Lelliottia amnigena, *Pantoea* spp., Pluralibacter gergoviae, *Raoultella* spp., *Serratia* spp. Total number of isolates of each organism: *Citrobacter* spp. = 1,950; E. cloacae = 3,864; Enterobacter spp. = 1,179; E. coli = 11,728; K. aerogenes = 1,289; K. pneumoniae = 10,157; Klebsiella spp. = 2,375; M. morganii = 1,294; Proteus spp. = 2,403; *Providencia* spp. = 959; S. marcescens = 2,055; Others = 115. (B) Distribution of organisms among meropenem-nonsusceptible *Enterobacterales* (N = 2,228). *Does not include isolates of E. cloacae; **Does not include isolates of K. aerogenes and K. pneumoniae; Total number of isolates of each organism: *Citrobacter* spp. = 55; E. cloacae = 193; Enterobacter spp. = 24; E. coli = 148; K. aerogenes = 25; K. pneumoniae = 1,592; Klebsiella spp. = 38; M. morganii = 8; Proteus spp. = 18; *Providencia* spp. = 64; S. marcescens = 63.

A total of 39,368 *Enterobacterales* isolates were collected from 55 countries in 2018 (*n* = 19,659) and 2019 (*n* = 19,709; Fig. 1; Table S1 and S2). Due to the collection quotas for the study, the majority of isolates belonged to Escherichia coli (%/N; 29.8%/11,728) and Klebsiella pneumoniae (25.8%/10,157; Fig. 2A; Table S2).

**FIG 3:**
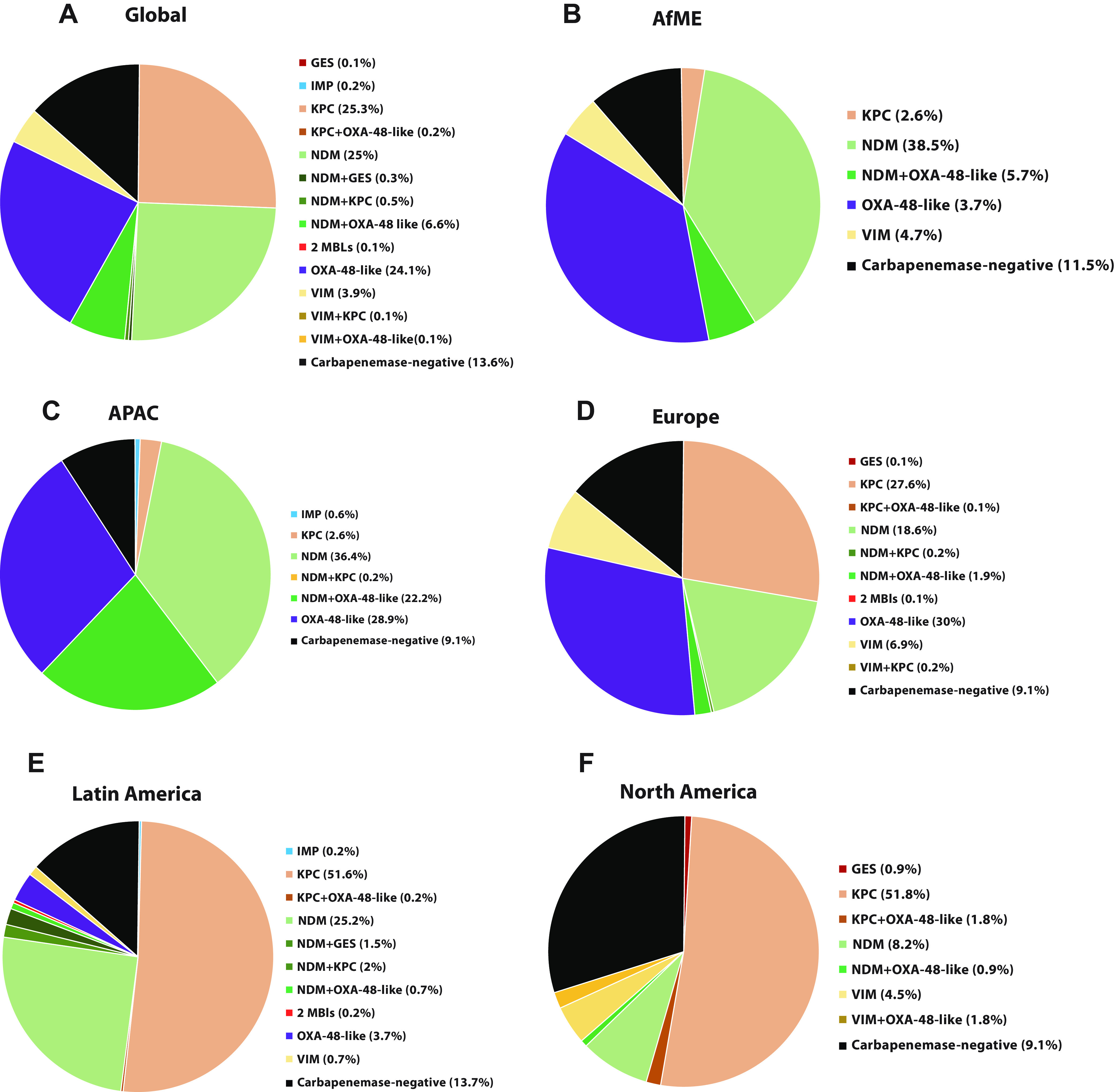
Distribution of carbapenem resistance mechanisms identified in meropenem-nonsusceptible *Enterobacterales* isolates collected globally and across different regions in 2018 and 2019. (A) Meropenem-nonsusceptible isolates collected globally in 2018 and 2019 (N = 2,228). (B) Isolates collected in Africa and Middle East (N = 192). (C) Isolates collected in Asia Pacific (N = 505). (D) Isolates collected in Europe (N = 1020). (E) Isolates collected in Latin America (N = 401). (F) Isolates collected in North America (N = 110). AfME, Africa and Middle East; APAC, Asia Pacific; NDM, New Delhi metallo-β-lactamase; KPC, Klebsiella pneumoniae carbapenemase; OXA, oxacillinase; VIM, Verona integron-encoded metallo-β-lactamase; GES, Guiania extended-spectrum β-lactamase; VEB, Vietnamese extended-spectrum β-lactamase; MBL, Metallo-β-lactamase.

Globally, 2,228 isolates (5.7%) were MEM-NS. Among the different geographical regions, isolates collected in Asia/Pacific (APAC) had the highest proportion (8.4%; n/N, 505/6,007), and those collected in North America had the lowest proportion of meropenem-nonsusceptibility (1.9%, 110/5,728; Fig. 1; Table S1). Among MEM-NS isolates collected, 71.5% were Klebsiella pneumoniae (1,592/2,228) with smaller proportions of other species accounting for the resistant isolates in the study such as: Enterobacter cloacae (8.7%, 193/2,228) and Escherichia coli (6.6%, 148/2,228). The remaining 13.2% nonsusceptible isolates included *Providencia* spp., Serratia marcescens, Klebsiella spp., *Citrobacter* spp., Enterobacter spp., Proteus spp., and Morganella morganii (Fig. 2B). The majority of carbapenem resistance mechanisms identified among MEM-NS isolates collected globally were MBLs (36.7%, 818/2,228), while comparable percentages of isolates carried KPC (25.5%, 568/2,228) or OXA-48-like β-lactamases (24.1%, 538/2,228). Among the MBL genes identified in these isolates, the majority encoded variants of NDM (88.4%, 723/818), while those encoding variants of VIM accounted for 11.1% (91/818) and those encoding variants of IMP accounted for 0.5% (4/818). Less than 0.1% (2/2228) of the MEM-NS isolates carried a GES carbapenemase. No carbapenemase genes were detected in 13.6% (302/2,203) of the MEM-NS isolates (Fig. 3A; Table 1). The distribution of resistance mechanisms observed among MEM-NS isolates varied across regions (Fig. 3B to F).

**TABLE 1:** Distribution of carbapenemase variants among meropenem-nonsusceptible, carbapenemase-positive *Enterobacterales* isolates collected in 2018 and 2019

	KPC		OXA-48-like		NDM		VIM		IMP	GES	No carbapenemase	
	2018	2019	2018	2019	2018	2019	2018	2019	2019	2019	2018	2019
Global												
Klebsiella pneumoniae	KPC-2 (91) KPC-3 (90) KPC-31 (1) KPC-46 (1) KPC-TYPE^*a*^ (6)	KPC-2 (154) KPC-3 (134) KPC-4 (1) KPC-66 (1) KPC-TYPE^*a*^ (1)	OXA-48 (138) OXA-181 (16) OXA-232 (43) OXA-244 (5) OXA-370 (1)	OXA-48 (144) OXA-181 (18) OXA-232 (105) OXA-48-TYPE (1)	NDM-1 (130) NDM-4 (1) NDM-5 (33) NDM-7 (4) NDM-9 (2) NDM-19 (1) NDM-TYPE (5)	NDM-1 (192) NDM-5 (67) NDM-6 (2) NDM-7 (12) NDM-9 (3)	VIM-1 (7) VIM-19 (2)	VIM-1 (11) VIM-4 (5)	IMP-1 (1) IMP-26 (2)	GES-5 (1)	76	84
Enterobacter cloacae	KPC-2 (4) KPC-3 (6) KPC-6 (1)	KPC-2 (13) KPC-3 (1)	OXA-48 (9) OXA-181 (4)	OXA-48 (10) OXA-181 (1)	NDM-1 (34) NDM-5 (2) NDM-7 (2) NDM-16 (1)	NDM-1 (37) NDM-5 (3) NDM-6 (1) NDM-7 (3)	VIM-1 (18) VIM-4 (6)	VIM-1 (13)	IMP-4 (1)		16	7
Escherichia coli	KPC-2 (2) KPC-3 (1)	KPC-2 (5) KPC-3 (6)	OXA-48 (3) OXA-181 (1) OXA-232 (1)	OXA-48 (4) OXA-162 (1) OXA-181 (2) OXA-232 (2)	NDM-1 (5) NDM-5 (28) NDM-7 (1)	NDM-1 (9) NDM-4 (1) NDM-5 (41)	VIM-4 (1)	VIM-1 (2) VIM-4 (1)			21	10
*Providencia* spp.				OXA-48 (2)	NDM-1 (14) NDM-4 (1)	NDM-1 (28) NDM-5 (1) NDM-7 (1)	VIM-1 (3) VIM-4 (1)		IMP-27 (1)		9	3
Serratia marcescens	KPC-2 (5) KPC-3 (3)	KPC-2 (5) KPC-3 (1)	OXA-48 (2)	OXA-48 (3)	NDM-1 (4) NDM-5 (2)	NDM-1 (7) NDM-7 (1)	VIM-1 (3)	VIM-1 (1)			18	8
*Citrobacter* spp.	KPC-2 (2)	KPC-2 (11) KPC-3 (1)	OXA-48 (3) OXA-162 (1) OXA-181 (1)	OXA-48 (5)	NDM-1 (6) NDM-7 (1)	NDM-1 (11)	VIM-1 (3) VIM-24 (1) VIM-4 (2)	VIM-1 (2)			4	1
Klebsiella spp.	KPC-2 (3) KPC-3 (2)	KPC-2 (6) KPC-3 (5)	OXA-48 (3)	OXA-48 (2)	NDM-1 (2)	NDM-1 (2) NDM-7 (1) NDM-9 (1)	VIM-1 (2)	VIM-1 (2)			4	3
Klebsiella aerogenes	KPC-2 (1)		OXA-48 (2)		NDM-1 (1)						14	7
Enterobacter spp.	KPC-2 (1)	KPC-2 (2)		OXA-48 (5)		NDM-1 (4)	VIM-1 (2) VIM-23 (1) VIM-5 (1)	VIM-1 (1)		GES-6 (1)	2	4
Proteus spp.					NDM-1 (1) NDM-6 (2)	NDM-1 (4) NDM-5 (1)					6	4
Morganella morganii		KPC-2 (1)			NDM-1 (4)	NDM-1 (1) NDM-6 (1)					1	
AfME												
Klebsiella pneumoniae	KPC-2 (1) KPC-3 (1)	KPC3 (2)	OXA-48 (10) OXA-181 (5) OXA-232 (1)	OXA-48 (15) OXA-181 (8) OXA-232 (12)	NDM-1 (13) NDM-7 (3)	NDM-1 (28) NDM-5 (5) NDM-7 (7)	VIM-1 (1)	VIM-1 (1)			9	7
Enterobacter cloacae		KPC-2 (1)	OXA-48 (6) OXA-181 (4)	OXA-48 (3) OXA-181 (1)	NDM-1 (4) NDM-7 (1)	NDM-1 (1) NDM-7 (1)	VIM-4 (4)				1	
Escherichia coli				OXA-48 (1)	NDM-5 (1)	NDM-1 (1) NDM-5 (2)	VIM-4 (1)	VIM-1 (1) VIM-4 (1)			1	
*Providencia* spp.					NDM-1 (4) NDM-4 (1)	NDM-1 (5) NDM-7 (1)						
Serratia marcescens				OXA-48 (2)	NDM-1 (1)	NDM-1 (2) NDM-7 (1)					2	
*Citrobacter* spp.			OXA-181 (1)	OXA-48 (1)	NDM-1 (1)	NDM-1 (1)						
Enterobacter spp.				OXA-48 (1)								
Proteus spp.												1
Morganella morganii					NDM-1 (1)							
APAC												
Klebsiella pneumoniae	KPC-2 (5)	KPC-2 (7) KPC-4 (1)	OXA-48 (1) OXA-181 (8) OXA-232 (39)	OXA-48 (2) OXA-181 (9) OXA-232 (80) OXA-48-TYPE (1)	NDM-1 (25) NDM-4 (1) NDM-5 (6) NDM-7 (1) NDM-9 (1) NDM-TYPE (1)	NDM-1 (44) NDM-5 (20) NDM-7 (5) NDM-9 (2)			IMP-26 (2)		13	7
Enterobacter cloacae					NDM-1 (12) NDM-7 (1)	NDM-1 (17) NDM-5 (2) NDM-7 (1)			IMP-4 (1)		3	
Escherichia coli			OXA-181 (1) OXA-232 (1)	OXA-181 (1) OXA-232 (2)	NDM-1 (1) NDM-5 (21) NDM-7 (1)	NDM-1 (3) NDM-4 (1) NDM-5 (33)					8	3
*Providencia* spp.					NDM-1 (1)	NDM-1 (14)					1	
Serratia marcescens					NDM-1 (1)	NDM-1 (4)					3	2
*Citrobacter* spp.				OXA-48 (1)	NDM-1 (2) NDM-7 (1)	NDM-1 (2)					1	
Klebsiella spp.					NDM-1 (2)	NDM-1 (2) NDM-7 (1) NDM-9 (1)					1	
Klebsiella aerogenes											1	
Enterobacter spp.												1
Proteus spp.					NDM-1 (1)	NDM-1 (1) NDM-5 (1)					2	
Morganella morganii					NDM-1 (1)	NDM-1 (1)						
Europe												
Klebsiella pneumoniae	KPC-2 (32) KPC-3 (68) KPC-31 (1) KPC-46 (1)	KPC-2 (62) KPC-3 (106) KPC-66 (1)	OXA-48 (127) OXA-181 (3) OXA-232 (3) OXA-244 (5)	OXA-48 (124) OXA-181 (1) OXA-232 (4)	NDM-1 (83) NDM-5 (3) NDM-TYPE (1)	NDM-1 (72) NDM-5 (2)	VIM-1 (4) VIM-19 (2)	VIM-1 (10) VIM-4 (5)			43	39
Enterobacter cloacae	KPC-2 (2) KPC-3 (1)	KPC-3 (1)	OXA-48 (3)	OXA-48 (7)	NDM-1 (8) NDM-5 (2) NDM-16 (1)	NDM-1 (9)	VIM-1 (17) VIM-4 (2)	VIM-1 (11)			7	4
Escherichia coli	KPC-3 (1)	KPC-3 (4)	OXA-48 (3)	OXA-48 (7) OXA-162 (1) OXA-181 (1)	NDM-1 (1) NDM-5 (6)	NDM-1 (1) NDM-5 (3)		VIM-1 (1)			6	6
*Providencia* spp.				OXA-48 (2)	NDM-1 (7)	NDM-1 (3)	VIM-1 (3) VIM-4 (1)				7	3
Serratia marcescens			OXA-48 (2)	OXA-48 (1)	NDM-1 (2) NDM-5 (2)		VIM-1 (2)	VIM-1 (1)			8	2
*Citrobacter* spp.	KPC-2 (1)		OXA-48 (1) OXA-162 (1)	OXA-48 (3)		NDM-1 (2)	VIM-1 (3) VIM-4 (2)	VIM-1 (2)			1	1
Klebsiella spp.		KPC-3 (2)	OXA-48 (3)	OXA-48 (2)			VIM-1 (1)	VIM-1 (1)			1	2
Klebsiella aerogenes			OXA-48 (2)								4	4
Enterobacter spp.				OXA-48 (4)		NDM-1 (1)	VIM-1 (2) VIM-5 (1)	VIM-1 (1)		GES-6 (1)	1	2
Proteus spp.						NDM-1 (1)					4	1
Morganella morganii					NDM-1 (1)							
Latin America												
Klebsiella pneumoniae	KPC-2 (43) KPC-3 (14) KPC-TYPE (6)	KPC-2 (82) KPC-3 (19) KPC-TYPE (1)	OXA-370 (1)	OXA-48 (3) OXA-232 (9)	NDM-1 (9) NDM-9 (1) NDM-TYPE (3)	NDM-1 (46) NDM-5 (2) NDM-6 (2) NDM-9 (1)	VIM-1 (1)		IMP-1 (1)		8	28
Enterobacter cloacae	KPC-2 (2)	KPC-2 (11)			NDM-1 (9)	NDM-1 (9) NDM-6 (1)					3	1
Escherichia coli	KPC-2 (2)	KPC-2 (5)			NDM-1 (2)	NDM-1 (4) NDM-5 (3)					3	1
*Providencia* spp.					NDM-1 (2)	NDM-1 (6) NDM-5 (1)			IMP-27 (1)		1	
Serratia marcescens	KPC-2 (4)	KPC-2 (5)				NDM-1 (1)					3	2
*Citrobacter* spp.	KPC-2 (1)	KPC-2 (4)	OXA-48 (2)		NDM-1 (3)	NDM-1 (3)	VIM-24 (1)					
Klebsiella spp.		KPC-2 (4) KPC-3 (1)										
Klebsiella aerogenes	KPC-2 (1)				NDM-1 (1)						2	1
Enterobacter spp.	KPC-2 (1)	KPC-2 (2)				NDM-1 (3)	VIM-23 (1)					
Proteus spp.					NDM-6 (2)	NDM-1 (2)						2
Morganella morganii					NDM-1 (1)	NDM-6 (1)						
North America												
Klebsiella pneumoniae	KPC-2 (10) KPC-3 (7)	KPC-2 (3) KPC-3 (7)				NDM-1 (2)	VIM-1 (1)				3	3
Enterobacter cloacae	KPC-3 (5) KPC-6 (1)	KPC-2 (1)			NDM-1 (1)	NDM-1 (1) NDM-5 (1) NDM-7 (1)	VIM-1 (1)	VIM-1 (2)			2	2
Escherichia coli		KPC-3 (2)			NDM-1 (1)						3	
Serratia marcescens	KPC-2 (1) KPC-3 (1)	KPC-3 (1)					VIM-1 (1)				2	2
*Citrobacter* spp.		KPC-2 (7) KPC-3 (1)				NDM-1 (3)					1	
Klebsiella spp.	KPC-2 (3) KPC-3 (2)	KPC-2 (2) KPC-3 (2)					VIM-1 (1)	VIM-1 (1)			2	1
Klebsiella aerogenes											7	2
Enterobacter spp.											1	1
Morganella morganii		KPC-2 (1)									1	

a Type is appended when the full coding sequence could not be amplified by PCR, thus no variant was determined.

Among MEM-NS isolates collected in Africa and the Middle East (AfME), MBLs were the most frequently identified carbapenemases (49%, 94/192), followed by OXA-48-like β-lactamases (42.7%, 82/192). The majority of the MBL-positive isolates carried NDM β-lactamases (90.4%, 85/94), while VIM was the only other MBL identified, in a relatively small proportion of MBL-positive isolates (4.7%, 9/94; Fig. 3B).

Among those collected in APAC, the majority of the MEM-NS isolates characterized carried MBLs (59.4%, 300/505), followed by OXA-48-like β-lactamases (51.1%, 258/505). Similar to global proportions, NDM (99%, 297/300) was predominant among MBL genes identified among isolates collected in APAC. IMP was the only other MBL identified in isolates collected in APAC (Fig. 3C).

Among the MEM-NS isolates collected in Europe, the most frequently identified carbapenemase genes encoded OXA-48-like β-lactamases (32.0%, 326/1,020) and a similar proportion of isolates carried MBLs (27.8%, 284/1,020) and KPC-type carbapenemases (28.0%, 286/1,020). No IMP-positive isolates were detected among those collected in Europe (Fig. 3D).

Among the MEM-NS isolates collected in Latin America and North America, KPC was the most frequently identified carbapenemase in 53.9% (216/401) and 53.6% (59/110) of carbapenemase-positive isolates, respectively. MBLs accounted for a greater proportion of carbapenemases among the MEM-NS isolates collected in Latin America (30.7%, 123/401), than North America (15.5%,17/110). No isolates carrying IMP-type β-lactamases were detected among those collected in North America (Fig. 3E and F).

**FIG 4:**
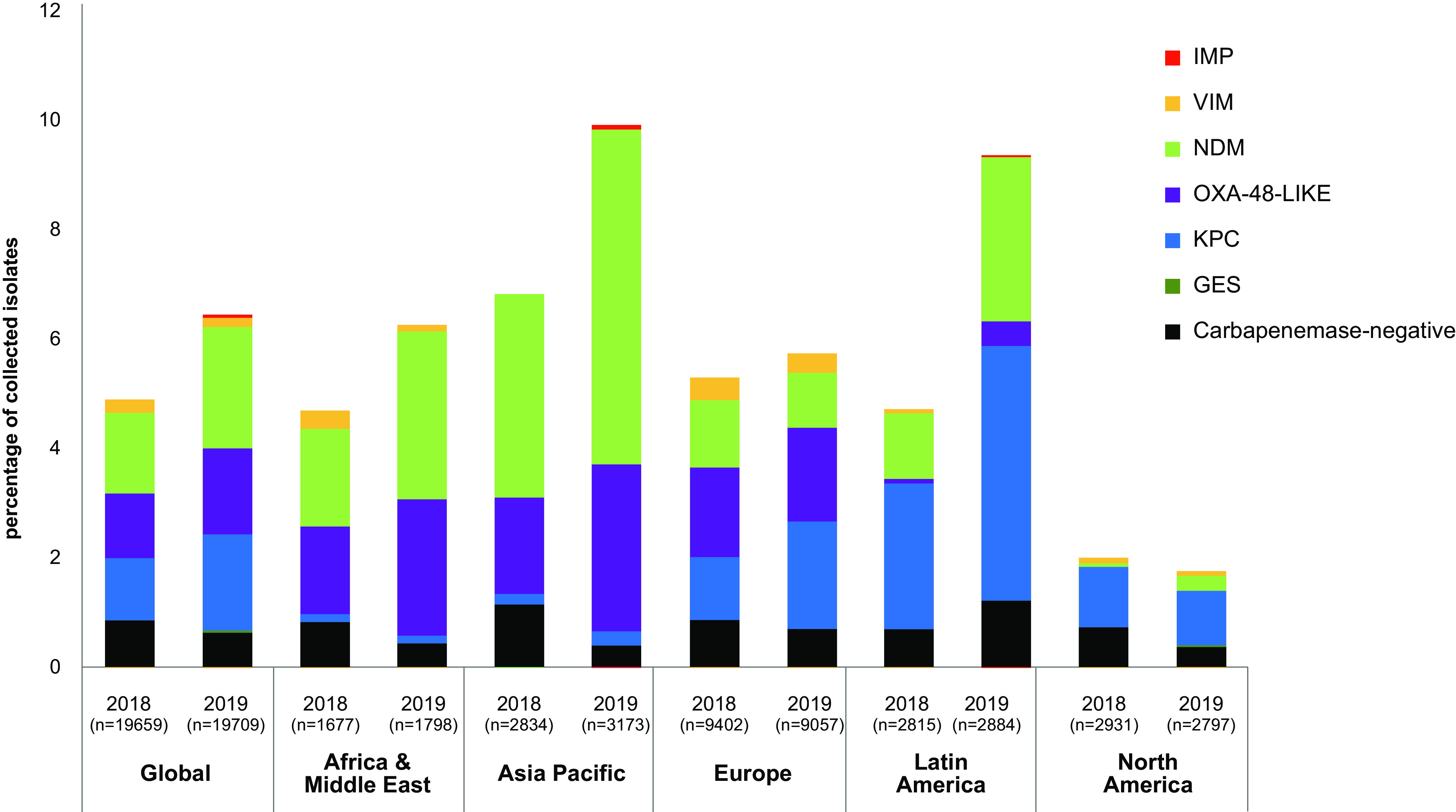
Proportion of carbapenem resistance mechanisms identified in meropenem-nonsusceptible *Enterobacterales* isolates collected globally and across different regions in 2018 and 2019. n, total number of isolates collected.

While the global proportion of meropenem-nonsusceptibility among isolates collected in 2018 versus 2019 increased from 4.9% to 6.4%, the proportion of carbapenemase families identified remained relatively consistent on the global scale. The year-over-year decrease in meropenem-susceptibility in this study, which was most stark among isolates collected in APAC and Latin America, correlated with increased detection of NDM-positive isolates in APAC, from 3.7% (104/2,834) to 6.1% (193/3,173) and Latin America, 1.2% (33/2,815) to 3.0% (86/2,884), as well as OXA-48-like-positive isolates in APAC, 3.1% (50/2,834) to 5.3% (96/3,173) and KPC-positive isolates collected in Latin America, 2.7% (74/2,815) to 4.8% (134/2,884; Fig. 4).

Globally, a total of 38 carbapenemase variants (nine NDM, eight KPC, eight OXA-48-like, six VIM, four IMP, and three GES) were identified among the MEM-NS isolates (Table 1 and 2). NDM-1 (68.7%, 497/723), KPC-2 (54.6%, 317/581), KPC-3 (43.4%, 252/581), OXA-48 (54.3%, 375/690), and VIM-1 (76.1%, 70/92) were the most commonly identified variants within their respective families (Table 2). NDM-1 was the predominant variant detected among NDM-positive isolates of all regions, except APAC which had a comparable proportion of isolates that carried NDM-5 (48.8%, 145/297) and NDM-1 (45.1%, 134/297; Table S4), noting that 78.1% of NDM-positive isolates collected in this region were collected in India (data not shown). KPC-2 accounted for the majority of KPC-type carbapenemases among MEM-NS isolates collected in APAC (92.9%, 13/14) and Latin America (80.6%, 174/216). KPC-3 was the most frequently identified variant of KPC within isolates collected in Europe (64.1%, 184/287) while similar proportions of isolates collected in North America carried KPC-2 (47.5%, 28/59) and KPC-3 (50.8%, 30/59; Table S4). OXA-48 was the most frequently identified variant of OXA-48-like β-lactamases among MEM-NS isolates collected in Europe (92.9%, 303/326) and AfME (61%, 50/82). OXA-232 was the most frequently identified OXA-48-like variant found among MEM-NS isolates collected in APAC (71.7%, 185/258). A similar proportion of OXA-48-like-positive isolates collected in Latin America carried OXA-48 (42.1%, 8/19) and OXA-232 (47.4%, 9/19). The majority of the OXA-48-like-positive isolates that carried OXA-181 were found among those collected in APAC (24.4%, 63/258) and AfME (23.2%, 19/82; Table S5). Globally, there was an increase in the proportion of OXA-232 relative to other variants of OXA-48-like (24% to 36.6%) from 2018 to 2019 (Table 2).

**TABLE 2:** Distribution of major NDM, KPC, and OXA-48-like variants in meropenem nonsusceptible *Enterobacterales* collected globally in 2018 and 2019

	2018	2019	Overall
NDM (N)	287	436	723
NDM-1 (n [% of N])	201 (70.0%)	296 (67.9%)	497 (68.7%)
NDM-5 (n [% of N])	65 (22.6%)	113 (25.9%)	178 (24.6%)
NDM (Others)^*a*^ (n [% of N])	21 (7.3%)	27 (6.2%)	48 (6.6%)
KPC (N)	223	358	581
KPC-2 (n [% of N])	112 (50.2%)	205 (57.3%)	317 (54.6%)
KPC-3 (n [% of N])	102 (45.7%)	150 (41.9%)	252 (43.4%)
KPC (Others)^*b*^ (n [% of N])	9 (4.0%)	3 (0.8%)	12 (2.1%)
OXA-48-Like (N)	288	402	690
OXA-48 (n [% of N])	175 (60.8%)	200 (49.8%)	375 (54.3%)
OXA-232 (n [% of N])	69 (24.0%)	147 (36.6%)	216 (31.3%)
OXA-181 (n [% of N])	37 (12.8%)	52 (12.9%)	89 (12.9%)
OXA-48-Like (Others)^*c*^ (n [% of N])	7 (2.4%)	3 (0.7%)	10 (1.4%)

a Includes NDM-7 (N = 26), NDM-6 (N = 6), NDM-9 (N = 6), NDM-TYPE (N = 5), NDM-4 (N = 3), NDM-16 (N = 1), NDM-19 (N = 1)

b Includes KPC-TYPE (N = 7), KPC-31 (N = 1), KPC-4 (N = 1), KPC-46 (N = 1), KPC-6 (N = 1), KPC-66 (N = 1)

c Includes OXA-244 (N = 5), OXA-162 (N = 2), OXA-163 (N = 1), OXA-370 (N = 1), OXA-48-TYPE (N = 1); N, total number of isolates collected; n, number of isolates of a particular variant.

Comparison of carbapenemase carriage across organisms revealed that isolates of K. pneumoniae accounted for the majority of KPC (84.5%, 480/568) and OXA-48-like (87.5%, 471/538) positive isolates globally. Although a large proportion of MBLs identified globally were carried by K. pneumoniae (58.9%, 480/816), they were identified in other species, including E. cloacae (14.8%, 121/816), E. coli (10.7%, 87/816), and *Providencia* spp. (6.1%, 18/816). Among the organisms carrying no identified carbapenemases, the majority of the isolates belonged to K. pneumoniae (53.0%, 160/302), followed by E. coli (10.3%, 31/302). Among the different geographical regions, majority of the OXA-48-like (95.9%, 140/146, Table 1) carbapenemases were carried by carbapenemase-positive isolates of K. pneumoniae.

Among all the MEM-NS isolates collected globally, 7.8% (173/2,228) carried two carbapenemases. The most frequently identified combination of carbapenemases was of NDM and OXA-48-like β-lactamases (84.4%, 146/173; Fig. 4A). Among the different geographical regions, the highest proportion of MEM-NS isolates carrying two carbapenemases were collected from APAC (22.4%, 113/505; Fig. 4).

## DISCUSSION

**FIG 5:**
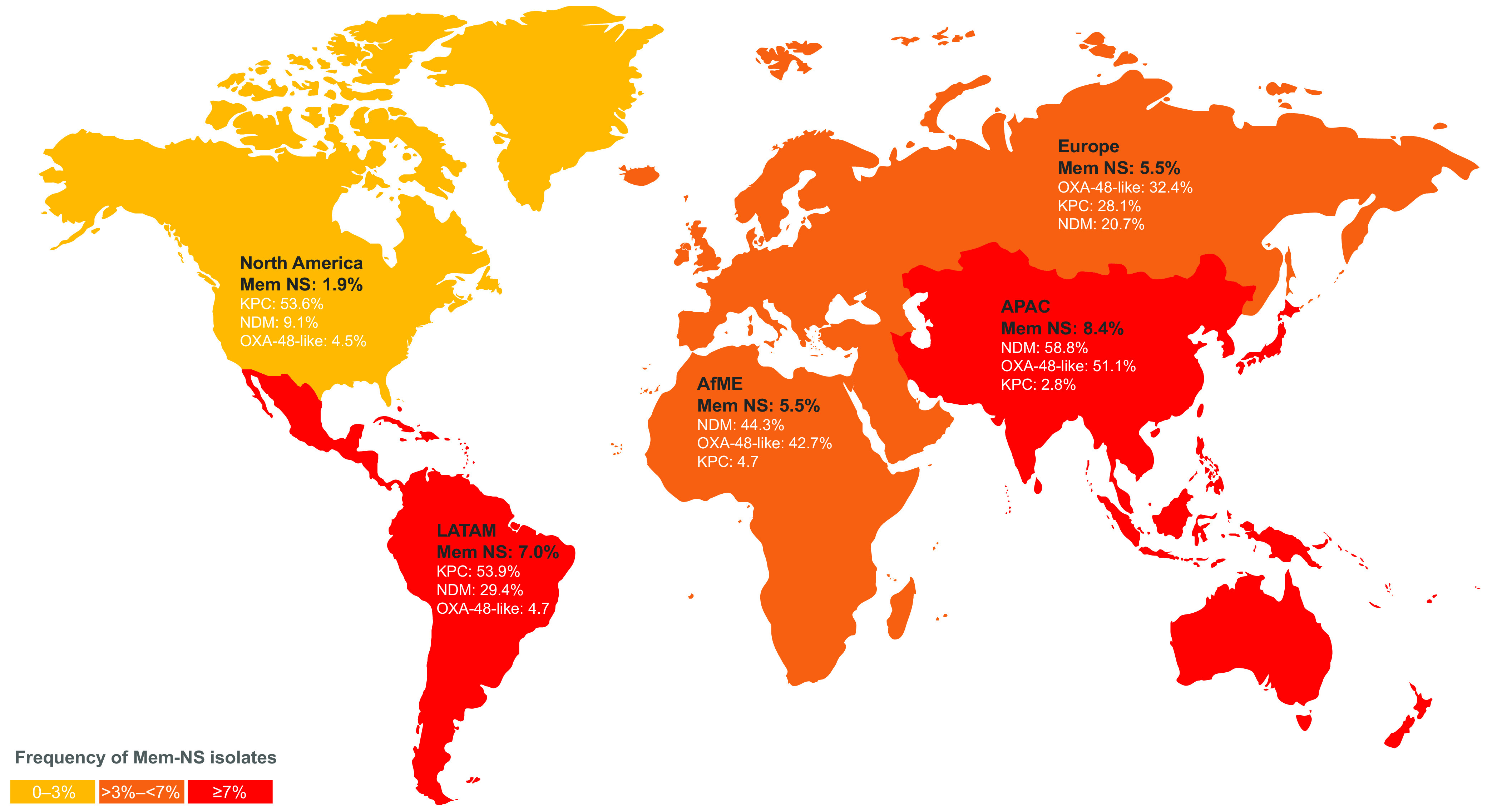
Frequency of meropenem nonsusceptibility among Enterobacterales isolates and associated carbapenemases, by region. AfME, Africa and Middle East; APAC, Asia/Pacific; Mem NS, Meropenem nonsusceptible, LATAM; Latin America. Countries contributing isolates from different regions included Israel, Jordan, Kuwait, Morocco, Nigeria, Qatar, Saudi Arabia, South Africa (Africa and Middle East); Australia, Hong Kong, India, Japan, South Korea, Malaysia, Philippines, Singapore, Taiwan, Thailand (Asia/Pacific); Belgium, Croatia, Czech Republic, France, germane, Greece, Hungary, Ireland, Italy, Latvia, Lithuania, Netherlands, Poland, Portugal, Romania, Russia, Spain, Switzerland, Turkey, Ukraine, United Kingdom (Europe); Argentina, Brazil, Chile, Columbia, Costa Rica, Guatemala, Mexico, Panama, Venezuela (Latin America); Canada and United States (North America).

This study evaluated the distribution of MEM-NS *Enterobacterales* and different families of carbapenemases among these isolates collected in 2018 and 2019. Overall, 5.7% of all *Enterobacterales* collected were MEM-NS. Of these, the majority (71.5%) were K. pneumoniae. Among carbapenemase-positive isolates, MBLs were present in 37.1%, KPC 25.5%, and OXA-48-like β-lactamases, 24.1%. NDM was the dominant MBL identified and carried by 88.4% of MBL-positive isolates. Among the different geographical regions, a large proportion of the MEM-NS isolates collected in AfME (49%), and APAC (59.4%) carried MBLs (Fig. 5). Among those collected in Europe, a comparable proportion of MEM-NS isolates carried OXA-48-like β-lactamases (32.0%), MBLs (27.8%), and KPC-type carbapenemases (28.0%; Fig. 5). The majority of MEM-NS isolates collected in Latin America (53.9%) and North America (53.6%) were KPC-positive (Fig. 5). Among the carbapenemases, the most frequently identified variants for each gene family encoded NDM-1 (68.7%), KPC-2 (54.6%), OXA-48 (54.3%, 375/690), and VIM-1 (76.1%, 70/92).

In the previous global surveillance study that assessed isolates collected in 2012 to 2017, 3.3% of the isolates were MEM-NS. Importantly, the previous study also reported an increase in the proportion of MEM-NS isolates from 2.7% in 2012 to 2014 to 3.8% in 2015 to 2017 (7). Our study additionally identified an increase in proportion of MEM-NS isolates from 4.9% in 2018 to 6.4% in 2019. Taken together, these results suggest that the global rise in carbapenem-nonsusceptibility among Enterobacterales continues at steady, if not accelerated pace.

Across all geographical regions, there was an increase in the proportion of MEM-NS isolates in this study compared to the previous study. In that previous study, in AfME, the number of MEM-NS isolates collected increased over 2.5 times (5.5% versus 1.97%) (7). The current study also identified a marked increase in the proportion of MEM-NS isolates from 2018 to 2019 across some regions: Latin America (4.7% versus 9.3%), APAC (6.8% versus 9.9%), and AfME (4.7% versus 6.3%; Fig. 4). Among isolates of species examined, the distribution of MEM-NS isolates in this study were in line with that of the previous study—K. pneumoniae (72.3% versus 76.7%, 2018 to 2019 versus 2012 to 2017), E. cloacae (8.8% versus 6.6%), and E. coli (6.7% versus 5.1%).

The majority of MEM-NS isolates assessed in this study carried MBLs (36.7%). This is in contrast to the previous study where the majority of the MEM-NS isolates carried KPC-type carbapenemase (47.4%) and only 20.6% carried MBLs (7). This increase in MBLs over the periods examined could be attributed to the marked increase in the proportion of MBL-positive isolates among the MEM-NS isolates in Latin America (2018 to 2019 versus 2012 to 2017: 30.7% versus 7.4%); North America (15.5% versus 4.9%), and Europe (27.8% versus 18.5%). The evident increase in the proportion of MBL-positive isolates among the MEM-NS isolates in the current study is consistent with several other studies that have also reported an increase and spread of MBLs globally and across different regions (1, 4, 11–13). Among all regions, the lowest frequency of MBL-positive isolates among the MEM-NS isolates was noted in North America in our study (15.5%), which is corroborated by another study (12). However, a marked increase in the frequency of MBLs among the MEM-NS isolates was also observed in North America from 2018 to 2019 in our study (10% versus 22%; Fig. 4). Overall, the proportion of MBL-positive isolates has been growing over the years which is of great concern due to their increasing resistance to carbapenems which were considered as the last-resort antimicrobials for treating infections (6). The majority of the MBLs in this study were identified in isolates of K. pneumoniae (58.9%), E. cloacae (14.8%), E. coli (10.2%), and *Providencia* spp. (6.1%). These data are in line with that of the previous study for K. pneumoniae (49.5%), E. cloacae (19.5%), and E. coli (6.2%; Table 1).

The previous study reported an increase in frequency of NDM-positivity, globally, and particularly in Latin America, Europe, and APAC, from 2012 to 2014 to 2015 to 2017 (6). Interestingly, the overall proportion of NDM-positive isolates among those that were MBL-positive, increased in this study compared to the previous study (2012 to 2017 versus 2018 to 2019: 61% versus 88.4%), suggesting a global spread of NDM carrying Enterobacterales (7). This is further supported by findings from other studies that have also indicated the increasing frequency of NDM carrying isolates worldwide (7, 14–16). In this study, NDM-positive isolates were detected in all regions and the highest proportion of these isolates were from APAC and AfME, similar to the previous study. Although the current study identified NDM-1 as the major NDM-variant, the proportion of isolates carrying NDM-1, among those that were NDM-positive, reduced compared to the previous study (2018 to 2019 versus 2012 to 2017: 68.7% versus 84.2%), and also from 2018 (70%) to 2019 (67.9%; Table 2). Notably, isolates carrying NDM-5 and NDM-7 were found mainly from AfME and APAC in this study which is corroborated by the previous study. Our study revealed an increase in proportion of these isolates, among those that were NDM-positive, compared to the previous study (AfME, 25.9% versus 17.6%; APAC 52.5% versus 31.3%) (7).

Comparable proportions of carbapenemase-positive isolates carried KPC-2 (50.2%) and KPC-3 (45.7%) in this study (Table 2). However, a much lower proportion of KPC-3 (30.7%) compared to KPC-2 (68.4%) was observed in the previous study (7). Interestingly, this trend of increasing frequency of KPC-3-positive isolates has been reported previously (16). Additionally, the current study revealed predominance of KPC-2 in Latin America (80.6%) and APAC (92.9%), while KPC-3 was predominant in Europe (64.1%). In the previous study, KPC-2 was the major variant observed in Europe (56.1%), Latin America (88.9%), and APAC (96.8%) (7).

In this study, the proportion of carbapenemase-positive isolates carrying OXA-48-like β-lactamases globally was comparable between 2018 and 2019. However, among the different geographical regions, an increase was noted only in the proportion of isolates collected in APAC and AfME from 2018 to 2019 (Fig. 4). Interestingly, Kazmierzack et al. also noted an increase in the proportion of OXA-48-like from 2012 to 2014 to 2015 to 2017 globally and in APAC and AfME (7). Among the different OXA-48-like variants identified in this study, OXA-48 was the most frequently identified, which was in line with the previous study (7). Of note, In the current study, there was a higher proportion of carbapenemase-positive isolates carrying OXA-232 and OXA-181 in APAC and AfME compared to other regions. Interestingly, the previous study also reported a similar trend. OXA-232 accounted for more of the OXA-48-like β-lactamases (31.3%) than did OXA-181 (12.9%) in the current study. However, in the past years, among the isolates that carried OXA-48-like carbapenemases, the proportion of isolates carrying OXA-181 had been reported to be more than those with OXA-232 (17). It should also be noted that OXA-232 carbapenemases hydrolyze carbapenem substrates less efficiently than either OXA-181 or OXA-48 (18). This overall increase in the proportion of OXA-232, among the MEM-NS isolates that carried OXA-48-like carbapenemases evident in the current study, could be driven by increasing frequency in APAC (71.7%), which is further supported by reports of outbreaks in that region since 2015 (17).

The proportion of isolates co-carrying two carbapenemases among those that were MEM-NS was 7.8% in this study (Fig. 3A), up from 2.7% reported in the previous study. This increase may be attributed to the increase in the proportion of isolates co-carrying MBLs and OXA-48-like carbapenemases in the current study (2% versus 6.7%, 2012 to 2017 versus 2018 to 2019). The increasing frequency of isolates with dual carbapenemases further complicates strategies to detect and overcome antimicrobial resistance.

This study has limitations. The data from this study cannot be used for epidemiological assessment or as a marker for prevalence of resistance mechanisms as each site collected a predefined number of isolates of given species. There were variations in the number of participating centers between years as well as the distribution of centers in each region. Changes in participating centers and countries must be taken into consideration when evaluating regional trends as well as comparing trends with previous studies and interpretations must be drawn cautiously. Additionally, carbapenemase carriage was only assessed in MEM-NS isolates which could have resulted in failure to identify some carbapenemases, especially among isolates carrying OXA-48-like or other carbapenemases with low activity that do not consistently confer resistance to meropenem and potentially biasing the results toward resistance mechanisms more specific to meropenem than carbapenems in general. Isolates collected from China were excluded in the study as they were characterized differently from the rest of the isolates. Notably, this study, but not the study performed previously, included isolates collected in India. Isolates collected in India comprised 23.4% of isolates collected in APAC for this study yet accounted for 75.8% of MEM-NS isolates for that region (data not shown), skewing the frequency of carbapenemase carriage toward trends in what may be specific to that country.

Despite the limitations, the data presented in this study provides crucial insights on the distribution of MEM-NS *Enterobacterales* and the carbapenemases that cause resistance among these organisms. The results of this study show an increasing trend of MEM-NS *Enterobacterales* globally with mechanisms of resistance varying across different regions. Among the MEM-NS isolates, the rise in the proportion of those carrying MBLs, particularly NDM, and those co-carrying different carbapenemases is of concern considering the increased resistance, paucity of treatment options, and the increased risk of mortality (1, 14, 19, 20). Hence, it is important to continuously monitor the trends of carbapenem resistance, both at global and regional levels. Understanding the trends in carbapenem-resistance mechanisms is important for the implementation of optimal stewardship programs, development of novel antimicrobial agents, and the allocation of public health resources, all of which are essential in the continued struggle against antimicrobial-resistant infections.

## MATERIALS AND METHODS

### Collection of isolates.

Nonduplicate clinical isolates (single isolate per patient) of *Enterobacterales*, independent of age, sex, previous antimicrobial use, or medical history were collected worldwide from patients in 2018 and 2019. Isolates were collected from 230 medical centers from 55 countries located in Africa and Middle East (AfME), Asia Pacific (APAC), Europe, Latin America, and North America. Isolates collected from mainland China were excluded from this study. Following identification, isolates were shipped to a central reference laboratory (International Health Management Associates, Inc. Schaumburg, IL, USA) for species confirmation using matrix-assisted laser desorption ionization-time of flight spectrometry (Bruker Biotyper MALDI-TOF, Bruker Daltonics, Billerica, MA, USA).

### Identification of isolates and detection of genes.

MICs of all organisms were determined using broth microdilution and antimicrobial susceptibility was interpreted according to Clinical and Laboratory Standards Institute (CLSI) guidelines (21, 22). *Enterobacterales* isolates testing as nonsusceptible to meropenem (MIC ≥ 2 μg/mL) were screened for the presence of β-lactamase genes encoding carbapenemases (KPC, OXA-48-like, GES, NDM, IMP, VIM, SPM, and GIM) and other β-lactamases (TEM, SHV, CTX-M-1 group, CTX-M-2 group, CTX-M-8 group, CTX-M-9 group, CTX-M-25 group, VEB, PER, ACC, ACT, CMY, DHA, FOX, MIR, and MOX) using five separate multiplex PCRs (*bla*_TEM_, *bla*_SHV_, *bla*_VEB_, *bla*_PER_, *bla*_GES_, and *bla*_OXA-48_; *bla*_IMP_, *bla*_VIM_, *bla*_SPM_, *bla*_NDM_, *bla*_KPC_, and *bla*_GIM_; *bla*_ACC_, *bla*_ACT_, *bla*_MIR_, *bla*_CMY_, *bla*_MOX_, *bla*_DHA_, and *bla*_FOX_; *bla*_CTX-M-1_, *bla*_CTX-M-2_, and *bla*_CTX-M-9_; *bla*_CTX-M-8_ and *bla*_CTX-M-25_), followed by amplification and sequencing of the full-length genes. The sequences were compared to sequences available in the National Center for Biotechnology Information Bacterial Antimicrobial Resistance Reference Gene Database (Bioproject 313047) (23). The protocol for PCR, amplification, and sequencing was as previously performed (24) using primers listed in Table S6.

## References

[1] van Duin D, Doi Y. 2017. The global epidemiology of carbapenemase-producing Enterobacteriaceae. Virulence 8:460–469. doi:10.1080/21505594.2016.1222343. 27593176 PMC5477705

[2] Zilahi G, Artigas A, Martin-Loeches I. 2016. What's new in multidrug-resistant pathogens in the ICU? Ann Intensive Care 6:96. doi:10.1186/s13613-016-0199-4. 27714706 PMC5053965

[3] Bush K. 2013. Carbapenemases: partners in crime. J Glob Antimicrob Resist 1:7–16. doi:10.1016/j.jgar.2013.01.005. 27873609

[4] Kazmierczak KM, de Jonge BLM, Stone GG, Sahm DF. 2020. Longitudinal analysis of ESBL and carbapenemase carriage among Enterobacterales and Pseudomonas aeruginosa isolates collected in Europe as part of the International Network for Optimal Resistance Monitoring (INFORM) global surveillance programme, 2013–17. J Antimicrob Chemother 75:1165–1173. doi:10.1093/jac/dkz571. 32040168

[5] Lasko MJ, Nicolau DP. 2020. Carbapenem-resistant Enterobacterales: considerations for treatment in the era of new antimicrobials and evolving enzymology. Curr Infect Dis Rep 22:6. doi:10.1007/s11908-020-0716-3. 32034524 PMC7223591

[6] Sheu CC, Chang YT, Lin SY, Chen YH, Hsueh PR. 2019. Infections caused by carbapenem-resistant enterobacteriaceae: an update on therapeutic options. Front Microbiol 10:80. doi:10.3389/fmicb.2019.00080. 30761114 PMC6363665

[7] Kazmierczak KM, Karlowsky JA, de Jonge BLM, Stone GG, Sahm DF. 2021. Epidemiology of carbapenem resistance determinants identified in meropenem-nonsusceptible enterobacterales collected as part of a global surveillance program, 2012 to 2017. Antimicrob Agents Chemother 65:e0200020. doi:10.1128/AAC.02000-20. 33972241 PMC8218680

[8] Logan LK, Weinstein RA. 2017. The epidemiology of carbapenem-resistant enterobacteriaceae: the impact and evolution of a global menace. J Infect Dis 215:S28–S36. doi:10.1093/infdis/jiw282. 28375512 PMC5853342

[9] Badarau A, Damblon C, Page MI. 2007. The activity of the dinuclear cobalt-beta-lactamase from Bacillus cereus in catalysing the hydrolysis of beta-lactams. Biochem J 401:197–203. doi:10.1042/BJ20061002. 16961465 PMC1698674

[10] Hirvonen VHA, Spencer J, van der Kamp MW. 2021. Antimicrobial resistance conferred by OXA-48 beta-lactamases: towards a detailed mechanistic understanding. Antimicrob Agents Chemother 65. doi:10.1128/AAC.00184-21. PMC8316048 33753332

[11] Garcia-Betancur JC, Appel TM, Esparza G, Gales AC, Levy-Hara G, Cornistein W, Vega S, Nunez D, Cuellar L, Bavestrello L, Castaneda-Mendez PF, Villalobos-Vindas JM, Villegas MV. 2021. Update on the epidemiology of carbapenemases in Latin America and the Caribbean. Expert Rev Anti Infect Ther 19:197–213. doi:10.1080/14787210.2020.1813023. 32813566

[12] Tan X, Kim HS, Baugh K, Huang Y, Kadiyala N, Wences M, Singh N, Wenzler E, Bulman ZP. 2021. Therapeutic options for metallo-beta-lactamase-producing enterobacterales. IDR 14:125–142. doi:10.2147/IDR.S246174. PMC7822077 33500635

[13] Tavoschi L, Forni S, Porretta A, Righi L, Pieralli F, Menichetti F, Falcone M, Gemignani G, Sani S, Vivani P, Bellandi T, Tacconi D, Turini L, Toccafondi G, Privitera G, Lopalco P, Baggiani A, Gemmi F, Luchini G, Petrillo M, Roti L, Pezzotti P, Pantosti A, Iannazzo S, Mechi MT, Rossolini GM, on Behalf of the Tuscan Clinical Microbiology Laboratory Network. 2020. Prolonged outbreak of New Delhi metallo-beta-lactamase-producing carbapenem-resistant Enterobacterales (NDM-CRE), Tuscany, Italy, 2018 to 2019. Euro Surveill 25. doi:10.2807/1560-7917.ES.2020.25.6.2000085. PMC7029447 32070467

[14] Khan AU, Maryam L, Zarrilli R. 2017. Structure, genetics and worldwide spread of New Delhi metallo-beta-lactamase (NDM): a threat to public health. BMC Microbiol 17:101. doi:10.1186/s12866-017-1012-8. 28449650 PMC5408368

[15] Wu W, Feng Y, Tang G, Qiao F, McNally A, Zong Z. 2019. NDM metallo-beta-lactamases and their bacterial producers in health care settings. Clin Microbiol Rev 32. doi:10.1128/CMR.00115-18. PMC6431124 30700432

[16] Castanheira M, Deshpande LM, Mendes RE, Canton R, Sader HS, Jones RN. 2019. Variations in the occurrence of resistance phenotypes and carbapenemase genes among enterobacteriaceae isolates in 20 years of the SENTRY antimicrobial surveillance program. Open Forum Infect Dis 6:S23–S33. doi:10.1093/ofid/ofy347. 30895212 PMC6419900

[17] Pitout JDD, Peirano G, Kock MM, Strydom KA, Matsumura Y. 2019. The global ascendency of OXA-48-type carbapenemases. Clin Microbiol Rev 33. doi:10.1128/CMR.00102-19. PMC6860007 31722889

[18] Potron A, Rondinaud E, Poirel L, Belmonte O, Boyer S, Camiade S, Nordmann P. 2013. Genetic and biochemical characterisation of OXA-232, a carbapenem-hydrolysing class D beta-lactamase from Enterobacteriaceae. Int J Antimicrob Agents 41:325–329. doi:10.1016/j.ijantimicag.2012.11.007. 23305656

[19] Boyd SE, Livermore DM, Hooper DC, Hope WW. 2020. Metallo-beta-lactamases: structure, function, epidemiology, treatment options, and the development pipeline. Antimicrob Agents Chemother 64. doi:10.1128/AAC.00397-20. PMC7508574 32690645

[20] Nordmann P, Poirel L. 2019. Epidemiology and diagnostics of carbapenem resistance in Gram-negative bacteria. Clin Infect Dis 69:S521–S528. doi:10.1093/cid/ciz824. 31724045 PMC6853758

[21] CLSI. 2018. Methods for dilution antimicrobial susceptibility tests for bacteria that grow aerobically; approved standard. 11th ed CLSI standard M07. Clinical and Laboratory Standards Institute, Wayne, PA.

[22] CLSI. 2020. Performance standards for antimicrobial susceptibility testing. 30th ed CLSI supplement M100. Clinical Laboratory Standards Institute, Wayne, PA.

[23] NCBI. Bacterial antimicrobial resistance reference gene database (Bioproject 313047). https://www.ncbi.nlm.nih.gov/bioproject/313047. Accessed 21 January 2022.

[24] Lob SH, Kazmierczak KM, Badal RE, Hackel MA, Bouchillon SK, Biedenbach DJ, Sahm DF. 2015. Trends in susceptibility of Escherichia coli from intra-abdominal infections to ertapenem and comparators in the United States according to data from the SMART program, 2009 to 2013. Antimicrob Agents Chemother 59:3606–3610. doi:10.1128/AAC.05186-14. 25801558 PMC4432174

