# Supplementary material for: Epidemiology of Resistance Determinants Identified in Meropenem-Nonsusceptible Enterobacterales Collected as Part of a Global Surveillance Study, 2018 to 2019
Source: Antimicrob Agents Chemother. 2023 Apr 19;67(5):e01406-22. doi: 10.1128/aac.01406-22 (PMC10190273; doi:10.1128/aac.01406-22)
Supplement: Supplemental file 1 — Supplemental material. Download aac.01406-22-s0001.pdf, PDF file, 0.1 MB [file aac.01406-22-s0001.pdf]

Epidemiology of Resistance Determinants Identified in Meropenem-non susceptible *Enterobacterales* collected as part of a Global Surveillance Program, 2018-2019

Mark Estabrook, Astrid Muyldermans, Daniel Sahm, Denis Pierard, Gregory Stone, and Eric Utt

Supplementary Data

Supplementary Table 1 Distribution of meropenem non-susceptible *Enterobacterales* collected in 2018 and 2019

	2018	2019	Overall
**Global**			
All *Enterobacterales* (N)	19659	19709	39368
Mem-NS *Enterobacterales* [n (% of N)]	964 (4.9%)	1264 (6.4%)	2228 (5.7%)
**Africa/Middle East**			
All *Enterobacterales* (N)	1677	1798	3475
Mem-NS *Enterobacterales* [n (% of N)]	79 (4.7%)	113 (6.3%)	192 (5.5%)
**Asia/pacific**			
All *Enterobacterales* (N)	2834	3173	6007
Mem-NS *Enterobacterales* [n (% of N)]	192 (6.8%)	313 (9.9%)	505 (8.4%)
**Europe**			
All *Enterobacterales* (N)	9402	9057	18459
Mem-NS *Enterobacterales* [n (% of N)]	500 (5.3%)	520 (5.7%)	1020 (5.5%)
**Latin America**			
All *Enterobacterales* (N)	2815	2884	5699
Mem-NS *Enterobacterales* [n (% of N)]	133 (4.7%)	268 (9.3%)	401 (7.0%)
**North America**			
All *Enterobacterales* (N)	2931	2797	5728
Mem-NS *Enterobacterales* [n (% of N)]	60 (2.0%)	50 (1.8%)	110 (1.9%)

Mem-NS, meropenem-nonsusceptible; N, total number of all Enterobacterales isolates collected; n, number of meropemem-nonsusceptible isolates

Supplementary Table 2. Distribution of different *Enterobacterales* collected globally in 2018 and 2019

	2018		2019		Overall	
Organisms n (% of N)	All isolates (N=19652)*	Mem-NS isolates (N=964)**	All isolates (N=19709)*	Mem-NS isolates (N=1264)**	All isolates (N=39368)*	Mem-NS isolates (N=2228)**
Klebsiella pneumoniae	4484 (22.8%)	653 (69%)	5673 (28.8%)	939 (74.3%)	10157 (25.8%)	1592 (71.5%)
Enterobacter cloacae	2378 (12.1%)	103 (10.9%)	1486 (7.5%)	90 (7.1%)	3864 (9.8%)	193 (8.7%)
Escherichia coli	5911 (30.1%)	64 (6.8%)	5817 (29.5%)	84 (6.6%)	11728 (29.8%)	148 (6.6%)
Providencia spp.	475 (2.4%)	28 (3%)	484 (2.5%)	36 (2.8%)	959 (2.4%)	64 (2.9%)
Serratia marcescens	1035 (5.3%)	37 (3.9%)	1020 (5.2%)	26 (2.1%)	2055 (5.2%)	63 (2.8%)
Citrobacter spp.	1001 (5.1%)	24 (2.5%)	948 (4.8%)	31 (2.5%)	1950 (5.0%)	55 (2.5%)
Klebsiella spp.	1076 (5.5%)	16 (1.7%)	1299 (6.6%)	22 (1.7%)	2375 (6.0%)	38 (1.7%)
Klebsiella aerogenes	825 (4.2%)	18 (1.9%)	464 (2.4%)	7 (0.6%)	1289 (3.3%)	25 (1.1%)
Enterobacter Spp.	637 (3.2%)	7 (0.7%)	542 (2.8%)	17 (1.3%)	1179 (3.0%)	24 (1.1%)
Proteus spp.	1131 (5.8%)	9 (1%)	1272 (6.5%)	9 (0.7%)	2403 (6.1%)	18 (0.8%)
Morganella morganii	649 (3.3%)	5 (0.5%)	645 (3.3%)	3 (0.2%)	1294 (3.3%)	8 (0.4%)
Others[1]	55 (0.3%)	0	60 (0.3%)	0	115 (0.3%)	0

Mem-NS, meropenem-nonsusceptible; [1]Includes *Cronobacter* spp. *Escherichia vulneris*, *Hafnia alvei*, Kosakonia *cowanii*, *Lelliottia amnigena*, *Pantoea* spp., *Pluralibacter gergoviae*, *Raoultella* spp., *Serratia* spp.; *% of all *Enterobacterales* for all isolates of each species; **% of all isolates of each species for meropenem-nonsusceptible isolates of that species.

Supplementary Table 3. Distribution of NDM variants among the meropenem-nonsusceptible *Enterobacterales* isolates collected across different regions in 2018 and 2019

	2018	2019	Overall
**Africa/Middle East**			
NDM (N)	30	55	85
NDM-1 [n (% of N)]	24 (80.0%)	38 (69.1%)	62 (72.9%)
NDM-5 [n (% of N)]	1 (3.3%)	7 (12.7%)	8 (9.4%)
NDM (Others)[1] [n (% of N)]	5 (16.7%)	10 (18.2%)	15 (17.6%)
**Asia/Pacific**			
NDM	104	193	297
NDM-1 [n (% of N)]	46 (44.2%)	88 (45.6%)	134 (45.1%)
NDM-5 [n (% of N)]	51 (49.0%)	94 (48.7%)	145 (48.8%)
NDM (Others)[2] [n (% of N)]	6 (5.8%)	10 (5.2%)	16 (5.4%)
**Europe**			
NDM (N)	118	94	212
NDM-1 [n (% of N)]	102 (86.4%)	89 (94.7%)	191 (90.1%)
NDM-5 [n (% of N)]	13 (11.0%)	5 (5.3%)	18 (8.5%)
NDM (Others)[3] [n (% of N)]	3 (2.5%)	0 (0.0%)	3 (1.4%)
**Latin America**			
NDM (N)	33	86	119
NDM-1 [n (% of N)]	27 (81.8%)	75 (87.2%)	102 (85.7%)
NDM-5 [n (% of N)]	0 (0.0%)	6 (7.0%)	6 (5.0%)
NDM (Others)[4] [n (% of N)]	6 (18.2%)	5 (5.8%)	11 (9.2%)
**North America**			
NDM (N)	2	8	10
NDM-1 [n (% of N)]	2 (100.0%)	6 (75.0%)	8 (80.0%)
NDM-5 [n (% of N)]	0 (0.0%)	1 (12.5%)	1 (10.0%)
NDM (Others)[5] [n (% of N)]	0 (0.0%)	1 (12.5%)	1 (10.0%)

[1]Includes NDM-4 (N=1), NDM-7 (N=14); [2]includes NDM-4 (N=2), NDM-7 (N=11), NDM-9 (N=4), NDM-TYPE (N=1); [3]includes NDM-TYPE (N=1) NDM-16 (N=1), NDM-19 (N=1); [4]includes NDM-6 (N=6), NDM-9 (N=2),

NDM-TYPE (N=3); [5]includes NDM-7 (N=1); N, number of NDM-positive isolates; n, number of isolates expressing that particular variant of NDM.

Supplementary Table 4. Distribution of KPC variants among the meropenem-nonsusceptible *Enterobacterales* isolates collected across different regions in 2018 and 2019

	2018	2019	Overall
**Africa/Middle East**			
KPC (N)	2	3	5
KPC-2 [n (% of N)]	1 (50.0%)	1 (33.3%)	2 (40.0%)
KPC-3 [n (% of N)]	1 (50.0%)	2 (66.7%)	3 (60.0%)
**Asia/Pacific**			
KPC (N)	5	9	14
KPC-2 [n (% of N)]	5 (100.0%)	8 (88.9%)	13 (92.9%)
KPC-3 [n (% of N)]	0 (0.0%)	0 (0.0%)	0 (0.0%)
KPC (Others)[1] [n (% of N)]	0 (0.0%)	1 (11.1%)	1 (7.1%)
**Europe**			
KPC (N)	107	180	287
KPC-2 [n (% of N)]	35 (32.7%)	65 (36.1%)	100 (34.8%)
KPC-3 [n (% of N)]	70 (65.4%)	114 (63.3%)	184 (64.1%)
KPC (Others)[2] [n (% of N)]	2 (1.9%)	1 (0.6%)	3 (1.0%)
**Latin America**			
KPC (N)	77	139	216
KPC-2 [n (% of N)]	57 (74.0%)	117 (84.2%)	174 (80.6%)
KPC-3 [n (% of N)]	14 (18.2%)	21 (15.1%)	35 (16.2%)
KPC (Others)[3] [n (% of N)]	6 (7.8%)	1 (0.7%)	7 (3.2%)
**North America**			
KPC (N)	32	27	59
KPC-2 [n (% of N)]	14 (43.8%)	14 (51.9%)	28 (47.5%)
KPC-3 [n (% of N)]	17 (53.1%)	13 (48.1%)	30 (50.8%)
KPC (Others)[4] [n (% of N)]	1 (3.1%)	0 (0.0%)	1 (1.7%)

KPC, *Klebsiella pneumoniae* carbapenemase; [1]includes KPC-4 (N=1); [2]includes KPC-31 (N=1), KPC-46 (N=1), KPC-66 (N=1); [3]includes KPC-TYPE (N=7); [4]includes KPC-6 (N=1); N, number of KPC-positive isolates; n, number of isolates expressing that particular variant of KPC.

Supplementary Table 5. Distribution of OXA-48-LIKE variants among the meropenem-nonsusceptible *Enterobacterales* isolates collected across different regions in 2018 and 2019

	**2018**	**2019**	**Overall**
**Africa/Middle East**			
OXA-48-Like (N)	30	52	82
OXA-48<br>[n (% of N)]	19<br>(63.3%)	31<br>(59.6%)	50<br>(61.0%)
OXA-232<br>[n (% of N)]	1<br>3.3%	12<br>23.1%	13<br>(15.9%)
OXA-181<br>[n (% of N)]	10<br>(33.3%)	9<br>(17.3%)	19<br>(23.2%)
**Asia/Pacific**			
OXA-48-Like (N)	89	169	258
OXA-48<br>[n (% of N)]	2<br>(2.2%)	7<br>(4.1%)	9<br>(3.5%)
OXA-232<br>[n (% of N)]	65<br>(73.0%)	120<br>(71.0%)	185<br>(71.7%)
OXA-181<br>[n (% of N)]	22<br>(24.7%)	41<br>(24.3%)	63<br>(24.4%)
OXA-48-Like (Others)[1]<br>[n (% of N)]	0<br>(0.0%)	1<br>(0.6%)	1<br>(0.4%)
**Europe**			
OXA-48-Like (N)	166	160	326
OXA-48<br>[n (% of N)]	152<br>(91.6%)	151<br>(94.4%)	303<br>(92.9%)
OXA-232<br>[n (% of N)]	3<br>(1.8%)	6<br>(3.8%)	9<br>(2.8%)
OXA-181<br>[n (% of N)]	5<br>(3.0%)	2<br>(1.3%)	7<br>(2.1%)
OXA-48-Like (Others)[2]<br>[n (% of N)]	6<br>(3.6%)	1<br>(0.6%)	7<br>(2.1%)
**Latin America**			
OXA-48-Like (N)	3	16	19
OXA-48<br>[n (% of N)]	2<br>(66.7%)	6<br>(37.5%)	8<br>(42.1%)
OXA-232<br>[n (% of N)]	0<br>(0.0%)	9<br>(56.3%)	9<br>(47.4%)
OXA-181<br>[n (% of N)]	0<br>(0.0%)	0<br>(0.0%)	0<br>(0.0%)
OXA-48-Like (Others)[3]<br>[n (% of N)]	1<br>(33.3%)	1<br>(6.3%)	2<br>(10.5%)
**North America**			
OXA-48-Like (N)	0	5	5

OXA-48<br>[n (% of N)]	0<br>(0.0%)	5<br>(100.0%)	5<br>(100.0%)
OXA-232<br>[n (% of N)]	0<br>(0.0%)	0<br>(0.0%)	0<br>(0.0%)
OXA-181<br>[n (% of N)]	0<br>(0.0%)	0<br>(0.0%)	0<br>(0.0%)

OXA, oxacillinase; [1]includes OXA-48-TYPE (N=1); [2]includes OXA-244 (N=5), OXA-162 (N=2); [3]includes OXA-163 (N=1), OXA-370 (N=1); N, number of OXA-48-like-positive isolates; n, number of isolates expressing that particular variant of OXA-48-like.

Supplementary Table 6. Primers used for multiplex PCR, gene amplification, and sequencing

Primer	Sequence (5' - 3')	Reference
**For *bla* gene detection by multiplex PCR**		
ACC-MF1	AACAGCCTCAGCAGCCGGTTA	(1)
ACC-MR1	TTCGCCGCAATCATCCCTAGC	
CIT-MF1 (CMY-II)	TGGCCAGAACTGACAGGCAAA	
CIT-MR1 (CMY-II)	TTTCTCCTGAACGTGGCTGGC	
CTX-M1-F21	AAAAATCACTGCGCCAGTTC	(2)
CTX-M1-R21	AGCTTATTCATCGCCACGTT	
CTX-M2-F21	CGACGCTACCCCTGCTATT	
CTX-M2-R21	CCAGCGTCAGATTTTTCAGG	
CTX-M8-A1	TCGCGTTAAGCGGATGATGC	
CTX-M25-A1	GCACGATGACATTCGGG	
CTX-M8/25-B1	AACCCACGATGTGGGTAGC	
CTX-M9-F21	CAAAGAGAGTGCAACGGATG	
CTX-M9-R21	ATTGGAAAGCGTTCATCACC	
DHA-MF1	AACTTTCACAGGTGTGCTGGGT	(1)
DHA-MR1	CCGTACGCATACTGGCTTTGC	
EBC-MF1(ACT/MIR)	TCGGTAAAGCCGATGTTGCGG	
EBC-MR1(ACT/MIR)	CTTCCACTGCGGCTGCCAGTT	
FOX-MF1	AACATGGGGTATCAGGGAGATG	
FOX-MR1	CAAAGCGCGTAACCGGATTGG	
GES-F1,2	AGTCGGCTAGACCGGAAAG	(3)
GES-R1,2	TTTGTCCGTGCTCAGGAT	
GIM-F1,2	TCGACACACCTTGGTCTGAA	(4)
GIM-R1,2	AACTTCCAACTTTGCCATGC	
IMP2-F1,2,3	GGAATAGAGTGGCTTAAYTCTC	
IMP2-R21,2,3	GGTTTAAYAAAACAACCACC	
KPCy-F1,2	TGTCACTGTATCGCCGTC	(5)
KPCy-R1,2	CTCAGTGCTCTACAGAAAACC	
MOX-MF1	GCTGCTCAAGGAGCACAGGAT	(1)
MOX-MR1	CACATTGACATAGGTGTGGTGC	
NDM-F1,2	CCGTATGAGTGATTGCGGCG	(6)
NDM-R1,2	GCCCAATATTATGCACCCGG	
OXA-24-F2	GGTTAGTTGGCCCCCTTAAA	(7)
OXA-24-R2	AGTTGAGCGAAAAGGGGATT	
OXA-48-F1	GCTTGATCGCCCTCGATT	(3)
OXA-48-R21,3	GATTTGCTSSGTRGCCGAAA	
PERpan-F1,2,3	TAGGYGTTGCMGTRTGGGG	Unpublished
PERpan-R1,2,3	GGTTTCRACCATCCAYTTCC	
SHV-51,2	CCTTTAAAGTAGTGCTCTGC	(8)

SHV-61,2	TTCGCTGACCGGCGAGTAGT	
SPM-F1,2	AAAATCTGGGTACGCAAACG	(9)
SPM-R1,2	ACATTATCCGCTGGAACAGG	
TEM-31,2	CATTTCCGTGTCGCCCTTATTC	(3)
TEM-41,2	CGTTCATCCATAGTTGCCTGAC	
VEB-F1,2	CATTTCCCGATGCAAAGCGT	
VEB-R1,2	CGAAGTTTCTTTGGACTCTG	
VIM-F1,2	GATGGTGTTTGGTCGCATA	(9)
VIM-R31,2	CGAATGCGCAGCACCAGGA	
**For *bla* gene amplification and sequencing**		
ACCfor-74	TCTTTTGCATGCGGATTGGC	Unpublished
ACCrev+1220	CCCCTGAGAAATCGGTGACT	
ACCint-seqF3	GAAGATGCGATTGAGMAAACS	
ACCint-seqR3	GGTTTKCTCAATCGCATCTTC	
preACT-F	CCGTTTGTCAGGCACAG	
preACT-R	CGCATAGGAGTTTCCTTACTG	
ACTint-seqR3	CGTTAATCCASGTATGGTCCAG	
ACTint-seqF3	CTGGACCATACSTGGATTAACG	
MIRint-seqF	CTCAAGCTGGACCATACCTG	
MIRint-seqR	GACCGATGCTGGCGTTAG	
MOX(a)for-119	TACCCACTTCACATCGGCTT	
MOX(a)rev+1175	ACCGAATGTACCGCCCTC	
MOX/CMY1int-seqF1	CAGCTCGGCGGATCTGC	
MOX/CMY1int-seqR13	GMGTACTGGCGATGGGA	
CMYIIfor-62	ACACACTGATTGCGTCTGAC	
CMYIIrev+11883	AAGAGMAAGAAAGGAGGYCC	
CMY2int-seqF	GCAATGACCAGACGCGTC	(8)
CMY2int-seqR	GACGCGTCTGGTCATTGC	
CTX-M1c-F	GACTATTCATGTTGTTGTTATTTC	(10)
CTX-M-1alt2-R3	TGAGTTYCCCCATTCCGTTT	Unpublished
CTX-M1-R2	AGCTTATTCATCGCCACGTT	(2)
CTX-M-1int Fseq	GACAGCTGGGAGACGAAACGTTC	(8)
preCTX-M-2-F	CAGGCTCAATTGTGGAGATATTGGC	Unpublished
preCTX-M-2-R	GACAAGACTGAAGTTCAGGAGCAC	
CTX-M2-R2	CCAGCGTCAGATTTTTCAGG	(2)
preCTX-M-9-F	TTGACCGTATTGGGAGTTTGAG	Unpublished
preCTX-M-9-R	GTGATCTGATCCTTCAACTCAGC	
CTX-M-9-R2	ATTGGAAAGCGTTCATCACC	(2)
CTX-M-9int Fseq	ATCGGCGATGAGACGTTTCG	Unpublished
CTXM8for-36	CTTCAGCCACACGGATTCAA	
CTXM8rev+901	GACAGAGCGCTCCACATTTT	
CTX-M-8-25B	AACCCACGATGTGGGTAGC	(2)
CTXM25for-50	CACGTGGAATTTAGGCTTCACT	Unpublished

CTXM25rev+945	TCGAAGAGAATAGCCAGGC	
CTX-M-25A	GCACGATGACATTCGGG	(2)
DHAfor-413	GATACTTGCCGCCGTYACTC	Unpublished
DHArev+1256	GTCAGTGCCCGATACTCTCA	
DHAint-seqF	GATGCCGTATGAGCAGTTGCT	
DHAint-seqR	CGTACGCATACTGGCTTTGC	
FOXfor-31	TGCCAATTTCATTCACCACGA	
FOXrev+11913	CCCCGGCGTMACWGTCAAAT	
FOXint-seqF	TGGTCACCGGTTTATCCG	
FOXint-seqR	GCAGGGTCTGGCTCATCA	
GES(green)for-41	AGACGGGCGTACAAAGATAAT	
GES(blue)rev(alt)+886	ACGAATTGTTAGACGGGCG	
GESint-seqR	CTCTCAGTAAGAGGTTAGTAGCC	
GESint-seqF	GGCTACTAACCTCTTACTGAGAG	
preIMP-F	GTTAGAAAAGGAAAAGTATG	(11)
preIMP-R	TGCTGCAACGACTTGTTAG	
IMP2-F3	GGAATAGAGTGGCTTAAYTCTC	(4)
IMPint-seqR	GAGAATTAAGCCACTCTATTCC	Unpublished
KPC (b)for-36	CGTCCGTAACTCCACCTTCA	
KPC (a)rev+1019	GCCATCGTCAGTGCTCTACA	
KPCint-Fseq	CGCCAATTTGTTGCTGAAGGAG	(8)
KPCint-Rseq	ACGTGGTATCGCCGATAGAGC	
NDMexgen-1F	TGCGGGGTTTTTAATGCTGA	Unpublished
NDMexgen-1R	ATGGCAGATTGGGGGTGAC	
preNDM-F	CACCTCATGTTTGAATTCGCC	(12)
preNDM-R	CTCTGTCACATCGAAATCGC	
NDMint-Fseq	CAGGACAAGATGGGCGGTATG	Unpublished
NDMint-Rseq	ACGCATTGGCATAAGTCGC	
OXA24/40for-31	GCCCCAAAATTTCCCCTAACA	
OXA24/40rev+8873	TTCGYATAASGYGTATTATGTTAA	
OXA-24-F	GGTTAGTTGGCCCCCTTAAA	(2)
OXA-24-R	AGTTGAGCGAAAAGGGGATT	
preOXA-48-A	TATATTGCATTAAGCAAGGG	
preOXA-48-B	CACACAAATACGCGCTAACC	(4)
OXA-48-F	GCTTGATCGCCCTCGATT	(3)
OXA-48-R	GATTTGCTCCGTGGCCGAAA	
PER(a)for-493	TTGGCTGCCTTTTGTATAATYCA	Unpublished
PER(a)rev+961	CCAGAGTCAGCGGCTTAGAT	
PER-505F3	GCAAATGAAGCRCARATGC	
PER-524R3	GCATYTGYGCTTCATTTGC	
SHV-1	CGCCGGGTTATTCTTATTTGTCGC	(13)
SHV-2	TCTTTCCGATGCCGCCGCCAGTCA	
SHVint-seqF	GATCGGCGACAACGTCAC	(8)

SHVint-seqR	GTGACGTTGTCGCCGATC	
SPMfor-53	CTTGATGGAGAAGTCGCTGC	Unpublished
SPMrev+878	TCGCGGCCTATGTTTGAAAG	
SPM-F	AAAATCTGGGTACGCAAACG	(9)
SPM-R	ACATTATCCGCTGGAACAGG	
TEMfor-483	AGACAATAACCCTGRTAAAT	Unpublished
TEMrev+935	CACCTAGATCCTTTTAAATT	
TEMint-seqF	GTGCTGCCATAACCATGAGTG	(8)
TEMint-seqR	CACTCATGGTTATGGCAGCAC	
preVEB-F2	AACCAGATAGGAGTACAGACATATG	Unpublished
preVEB-R2	GCCTATGAGCCAGTGTTAG	
VEBint-seqF	CGGAGGAACTGATTCTGTTC	
VEBint-seqR	GAACAGAATCAGTTCCTCCG	
preVIM-F	TATGCCGCACCCACCCCTATG	(14)
preVIM-clade1-R	GTTATGCCGCATCTGCTAC	Unpublished
VIM-F	GATGGTGTTTGGTCGCATA	(9)
VIM-R	CGAATGCGCAGCACCAG	
**For detection of 16S rDNA (internal reaction control)**		
U341F3	CCTACGGGRSGCAGCAG	(15)
16s-519r3	GWATTACCGCGGCKGCTG	(16)

References

1. Perez-Perez FJ, Hanson ND. 2002. Detection of plasmid-mediated AmpC beta-lactamase genes in clinical isolates by using multiplex PCR. J Clin Microbiol 40:2153-62.

2. Woodford N, Fagan EJ, Ellington MJ. 2006. Multiplex PCR for rapid detection of genes encoding CTX-M extended-spectrum (beta)-lactamases. J Antimicrob Chemother 57:154-5.

3. Dallenne C, Da Costa A, Decre D, Favier C, Arlet G. 2010. Development of a set of multiplex PCR assays for the detection of genes encoding important beta-lactamases in Enterobacteriaceae. J Antimicrob Chemother 65:490-5.

4. Poirel L, Walsh TR, Cuvillier V, Nordmann P. 2011. Multiplex PCR for detection of acquired carbapenemase genes. Diagn Microbiol Infect Dis 70:119-23.

5. Yigit H, Queenan AM, Anderson GJ, Domenech-Sanchez A, Biddle JW, Steward CD, Alberti S, Bush K, Tenover FC. 2001. Novel carbapenem-hydrolyzing beta-lactamase, KPC-1, from a carbapenem-resistant strain of Klebsiella pneumoniae. Antimicrob Agents Chemother 45:1151-61.

6. Lascols C, Hackel M, Marshall SH, Hujer AM, Bouchillon S, Badal R, Hoban D, Bonomo RA. 2011. Increasing prevalence and dissemination of NDM-1 metallo-beta-lactamase in India: data from the SMART study (2009). J Antimicrob Chemother 66:1992-7.

7. Woodford N, Ellington MJ, Coelho JM, Turton JF, Ward ME, Brown S, Amyes SG, Livermore DM. 2006. Multiplex PCR for genes encoding prevalent OXA carbapenemases in Acinetobacter spp. Int J Antimicrob Agents 27:351-3.

8. Lob SH, Kazmierczak KM, Badal RE, Hackel MA, Bouchillon SK, Biedenbach DJ, Sahm DF. 2015. Trends in susceptibility of Escherichia coli from intra-abdominal infections to ertapenem and comparators in the United States according to data from the SMART program, 2009 to 2013. Antimicrob Agents Chemother 59:3606-10.

9. Ellington MJ, Kistler J, Livermore DM, Woodford N. 2007. Multiplex PCR for rapid detection of genes encoding acquired metallo-beta-lactamases. J Antimicrob Chemother 59:321-2.

10. Mena A, Plasencia V, Garcia L, Hidalgo O, Ayestaran JI, Alberti S, Borrell N, Perez JL, Oliver A. 2006. Characterization of a large outbreak by CTX-M-1-producing Klebsiella pneumoniae and mechanisms leading to in vivo carbapenem resistance development. J Clin Microbiol 44:2831-7.

11. Hanson ND, Hossain A, Buck L, Moland ES, Thomson KS. 2006. First occurrence of a Pseudomonas aeruginosa isolate in the United States producing an IMP metallo-beta-lactamase, IMP-18. Antimicrob Agents Chemother 50:2272-3.

12. Kaase M, Nordmann P, Wichelhaus TA, Gatermann SG, Bonnin RA, Poirel L. 2011. NDM-2 carbapenemase in Acinetobacter baumannii from Egypt. J Antimicrob Chemother 66:1260-2.

13. Nuesch-Inderbinen MT, Hachler H, Kayser FH. 1996. Detection of genes coding for extended-spectrum SHV beta-lactamases in clinical isolates by a molecular genetic method, and comparison with the E test. Eur J Clin Microbiol Infect Dis 15:398-402.

14. Rodriguez-Martinez JM, Nordmann P, Fortineau N, Poirel L. 2010. VIM-19, a metallo-beta-lactamase with increased carbapenemase activity from Escherichia coli and Klebsiella pneumoniae. Antimicrob Agents Chemother 54:471-6.

15. Baker GC, Cowan DA. 2004. 16 S rDNA primers and the unbiased assessment of thermophile diversity. Biochem Soc Trans 32:218-21.

16. Lane DJ, Pace B, Olsen GJ, Stahl DA, Sogin ML, Pace NR. 1985. Rapid determination of 16S ribosomal RNA sequences for phylogenetic analyses. Proc Natl Acad Sci U S A 82:6955-9.
